# Blood cytopenias as manifestations of inherited metabolic diseases: a narrative review

**DOI:** 10.1186/s13023-024-03074-4

**Published:** 2024-02-14

**Authors:** Yannick Moutapam-Ngamby—Adriaansen, François Maillot, François Labarthe, Bertrand Lioger

**Affiliations:** 1 Service de Médecine Interne, CHRU de Tours, Tours Cedex 1, France; 2 Reference Center for Inborn Errors of Metabolism ToTeM, CHRU de Tours, Hôpital Clocheville, 49 Bd Béranger, 37000 Tours, France; 3 INSERM U1253, iBrain, Université François Rabelais de Tours, 10 Boulevard Tonnellé, 37000 Tours, France; 4 INSERM U1069, Nutrition, Croissance et Cancer, Faculté de Médecine, Université François Rabelais de Tours, 10 Boulevard Tonnellé, 37000 Tours, France; 5 Service de Pédiatrie, CHRU de Tours, Tours Cedex 1, France; 6 Service de Médecine Interne Et Polyvalente, 2, Centre Hospitalier de Blois, Mail Pierre Charlot, 41000 Blois, France

**Keywords:** Inherited metabolic diseases, Diagnosis, Cytopenia, Anemia, Neutropenia, Splenomegaly, Macrocytosis, Hemolysis

## Abstract

Inherited Metabolic Diseases (IMD) encompass a diverse group of rare genetic conditions that, despite their individual rarity, collectively affect a substantial proportion, estimated at as much as 1 in 784 live births. Among their wide-ranging clinical manifestations, cytopenia stands out as a prominent feature. Consequently, IMD should be considered a potential diagnosis when evaluating patients presenting with cytopenia. However, it is essential to note that the existing scientific literature pertaining to the link between IMD and cytopenia is limited, primarily comprising case reports and case series. This paucity of data may contribute to the inadequate recognition of the association between IMD and cytopenia, potentially leading to underdiagnosis. In this review, we synthesize our findings from a literature analysis along with our clinical expertise to offer a comprehensive insight into the clinical presentation of IMD cases associated with cytopenia. Furthermore, we introduce a structured diagnostic approach underpinned by decision-making algorithms, with the aim of enhancing the early identification and management of IMD-related cytopenia.

**Supplementary Information:**

The online version contains supplementary material available at 10.1186/s13023-024-03074-4.

## Introduction

Inherited metabolic diseases (IMD) are a group of genetic conditions mainly resulting from an enzyme deficiency, an enzymatic cofactor deficiency, or impaired transporter protein, leading to either accumulation or deficiency of a specific metabolite. Depending on the metabolite, it can either have a toxic effect on cellular processes after accumulation, induce an energy deficiency by decreased production, or cause structural abnormalities. With over 1400 IMD reported [1], the full range of clinical and biological manifestations has already been reported, and hematological manifestations are no exception. Thus, cytopenias, splenomegaly, monoclonal gammopathy, bleeding, coagulation abnormalities, and hematological malignancies are part of the spectrum of hematological manifestations associated with IMD [2].

IMD are part of a heterogeneous group of individually rare conditions. Still, their overall birth prevalence was as frequent as 1 in 784 live births in the United Kingdom, with an incidence of 40 cases per 100,000 live births [3, 4]. Epidemiological data on hematological manifestations of IMD, such as anemia, are scarce due to the low individual prevalence of each disease. Examples of well-defined epidemiology of hematological manifestations in IMD can be found in Gaucher disease [2], glucose-6-phosphate dehydrogenase (G6PD) deficiency [5], and pyruvate kinase deficiency [6]. For other IMD, the description of hematological findings is mainly based on case reports and case series.

Lack of awareness of hematological features associated with IMD, even among the prototype of hematological IMD such as Gaucher disease, has already been pointed out in previous studies. For example, only 20% of 406 hematology-oncology specialists considered Gaucher disease in the differential diagnosis for a patient with the classic symptoms, including cytopenia, hepatosplenomegaly, and bone pain [7]. Hematological abnormalities due to a metabolic disease are rarely seen in isolation and better knowledge of signs and symptoms suggestive of IMD could lead to shorter diagnostic delay. Based on our experience as a reference center for IMD in a tertiary hospital over 20 years and a literature review, we propose a narrative review of cytopenia associated with IMDs.

## Anemia

### Microcytic anemia

**Fig. 1:**
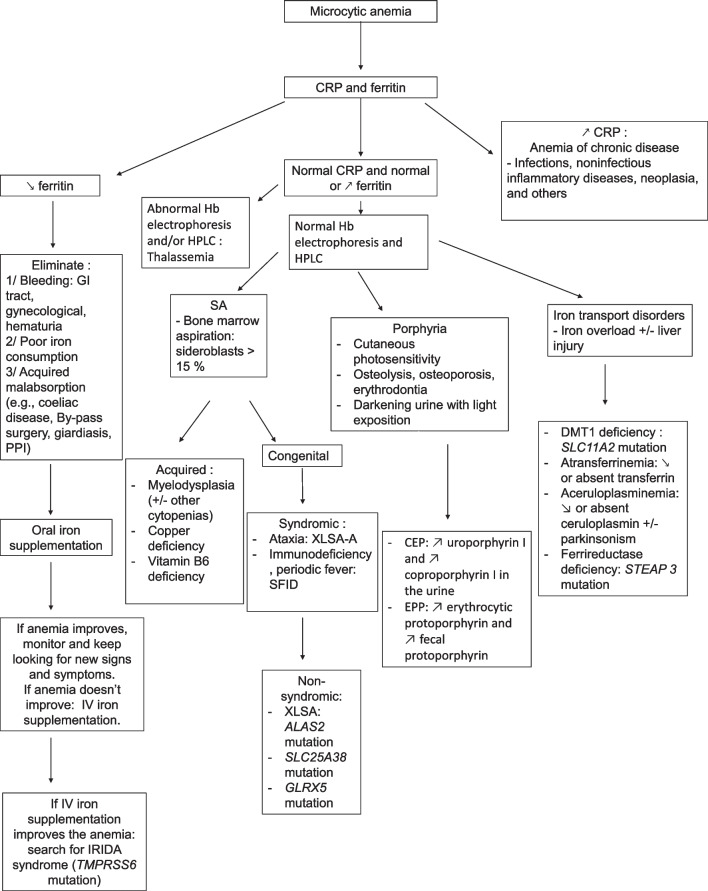
Microcytic anemia diagnostic pathway. CEP: congenital erythropoietic porphyria; CRP: C-reactive protein; EPP: erythropoietic protoporphyria; GI: gastro-intestinal; Hb: hemoglobin; HPLC: high-performance liquid chromatography; IRIDA: iron refractory iron deficiency anemia; IV: intravenous; PPI: proton pump inhibitor; SA: Sideroblastic anemia; SIFD congenital sideroblastic anemia-B-cell immunodeficiency-periodic fever-developmental delay syndrome; XLSA: X-linked sideroblastic anemia; XLSA-A: X-linked sideroblastic anemia with ataxia; ↗: increase; ↘: decreased

Microcytic anemia is characterized by the production of small-sized red blood cells due to a decreased production of hemoglobin. The most common causes of microcytic anemia are inflammation, iron deficiency, and thalassemia [1]. However, IMD can cause microcytic anemia through 2 primary mechanisms leading to reduced hemoglobin production: iron metabolism and heme production defects. The three main types of diseases leading to microcytic anemia are iron transport disorders, sideroblastic anemias, and porphyrias. They are summarized in Table 1, and a diagnostic algorithm is proposed in Fig. 1.

**Table 1:** IMD linked to microcytic anemia

Disease	Clinical signs	Biological signs	Gene (protein)	Diagnosis
Sideroblastic anemia (ring sideroblasts)				
XLSA	Liver cirrhosis and hepatocellular carcinoma	Iron overload + microcytic anemia	*ALAS2* (ALAS2)	ALAS2 mutation
SLC25A38	/	Isolated microcytic anemia	*SLC25A38* (SLC25A38)	SLC25A38 mutation
GLRX5	/ *		*GLRX5* (Glutaredoxin 5)	GLRX5 mutation
XLSA-A	Anemia before the age of 2, but ataxia can develop around 40 years	Microcytic or normocytic anemia	*ABCB7* (ABCB7)	ABCB7 mutation
SIFD	Recurrent infections, periodic fever, developmental delay	Microcytic anemia, hypogammaglobulinemia	*TRNT1* (tRNA nucleotidyl transferase 1)	TRNT1 mutation
[MLASA]^1^	Myopathy (exercise intolerance), ± cardiomyopathy, diabetes	Lactic acidosis	*PUS1* (tRNA pseudouridine synthase A), *YARS 2* (tyrosyl-tRNA synthetase 2), *MT-ATP6* (ATPase-6)	PUS1 mutation (if negative YARS 2 or MT-ATP6)
[Pearson marrow pancreas syndrome]^1^	Pancreatic dysfunction	Lactic acidosis	mtDNA deletion	mtDNA analysis
Porphyria				
CEP	Photosensitivity, corneal ulcer, skin atrophy, hypertrichosis, pink-red urine, erythrodontia	Hemolysis, basophilic stippling, macrocytic or microcytic anemia Urine: ↗ uroporphyrin I and coproporphyrin I Faeces: ↗ coproporphyrin I	*UROS* (uroporphyrinogen synthase) ± *GATA1* (GATA1) mutation if microcytic anemia	Uroporphyrinogen III synthase activity (< 15%)
EPP	Photosensitivity (erythema, edema, or isolated burning sensation but no blistering), Cholelithiasis, liver failure	Hepatic cytolysis, cholestasis, Plasma fluorescence peak at 634 nm testing	*FECH* (Ferrochelatase)	↗ protoporphyrin concentration in plasma, erythrocytes, and feces
Iron transport disorders				
DMT1 deficiency	Severe liver iron overload	↗ serum iron, normal total iron-TIBC, ↗ saturation of transferrin, slightly ↗ ferritin, and ↗ soluble transferrin receptor	*SLC11A2* (DMT1)	SLC11A2 mutation
Aceruloplasminemia	Triad: retinal degeneration, neuropsychiatric symptoms, and diabetes	↘ or undetectable ceruloplasmin, ↘ transferrin saturation, and ↗ serum ferritin,	*CP* (ceruloplasmin)	CP mutation
Atransferrinemia	Liver injury, heart failure, and Splenomegaly	↘↘ serum iron transferrin level: 0 or ↘↘	*TF* (transferrin)	TF mutation
IRIDA syndrome	Iron deficiency anemia with poor response to oral iron treatment	↘↘transferrin saturation ↘ low ferritin level	*TMPRSS6* (Matriptase 2)	TMPRSS6 mutation

CEP: congenital erythropoietic porphyria; EPP: erythropoietic protoporphyria; IMD (inherited metabolic diseases; IRIDA: iron refractory iron deficiency anemia; MLASA: myopathy with exercise intolerance, lactic acidosis and sideroblastic anemia; mtDNA: mitochondrial DNA SIFD: congenital sideroblastic anemia-B-cell immunodeficiency-periodic fever-developmental delay syndrome; TIBC: total iron binding capacity; XLSA: X-linked sideroblastic anemia; XLSA-A: X-linked sideroblastic anemia with ataxia; ↗: increased;↘: decreased; ↘↘: strongly reduced; /: no particular signs; []^1^ Causes of siderolastic anemia but macrocytic or normocytic

*There is a subtype of patients with GLRX5 mutation with neurological symptoms but without anemia

#### Iron transport disorders

Iron transport disorders leading to microcytic anemia are caused by impairment of one of the steps of transport of iron to the production site of heme inside the erythropoietic precursors. The leading causes are divalent metal transporter 1 (DMT1) deficiency, aceruloplasminemia, atransferrinemia, and IRIDA (Iron Refractory Iron Deficiency Anemia) syndrome.

##### IRIDA syndrome

First, reduced iron availability can be caused by reduced intestinal iron absorption, as in IRIDA syndrome. In this autosomal recessive condition, a mutation in the *TMPRSS6* gene coding for matriptase-2 causes an abnormally high level of hepcidin expression, a protein responsible for reducing iron absorption by promoting the degradation of ferroportin [9]. As a result, the affected patients have reduced enteric absorption of iron of variable penetrance, causing an iron deficiency anemia that can be supplemented by parenteral iron infusions rather than by oral route [10].

IRIDA syndrome should be considered in the context of low plasma ferritin and low transferrin saturation after negative investigations for digestive or gynecological bleeding. A lack of iron intake should also be considered a differential diagnosis, either due to a low iron diet or an iron absorption deficiency. Iron absorption can be impaired by excessive tea consumption or medications like proton pump inhibitors. Poor iron absorption can also be caused by malabsorption, as observed in coeliac disease. IRIDA syndrome should be suspected when no common cause is found and iron deficiency persists after adequate oral iron replenishment. In that case, parenteral iron administration should be provided, and if the microcytic anemia improves, genetic testing for mutations in TMPRSS6 should be done [11]. Transferrin saturation/hepcidin ratio could help diagnose this condition but is not readily available in every biochemistry laboratory [12].

##### DMT1 deficiency

In DMT1 deficiency, a mutation in the *SLC11A2* gene causes a lack of DMT1 expression [13]. Absorption of non-heme bound Fe2+ is impaired in the duodenum, as well as iron transport into the erythroid precursors. [14] Biologically, besides microcytic anemia, there is an iron overload with high serum ferritin and increased transferrin saturation that can lead to liver injury in severe cases [15].

##### Ferrireductase deficiency

Ferrireductase deficiency is caused by a mutation in the *STEAP3* gene. Lack of ferrireductase is responsible for a deficient reduction of Fe3+ that is bound to transferrin to Fe2+, a step needed for iron to pass through the DMT1 transporter to be able to join the site of heme synthesis in the red blood cells (RBC) precursors. This deficiency is responsible for severe microcytic anemia, and an association with gonadal deficiency has been described [16].

##### Aceruloplasminemia

Aceruloplasminemia is a deficit in functional ceruloplasmin, a protein that oxidizes Fe2+ into Fe3+, an essential step in incorporating iron into transferrin to transport iron to the bone marrow. A deficit in ceruloplasmin leads to an accumulation of iron in the extracellular and intracellular space of the liver, brain, retina, and pancreas. The transport to the bone marrow is inefficient, leading to microcytic anemia in 85% of patients, retinal degeneration, and diabetes mellitus. Ceruloplasmin deficiency also leads to a defect in copper transport and neurological symptoms such as ataxia, involuntary movements, Parkinson’s syndrome, and cognitive dysfunction [17]. Biologically, aceruloplasminemia is associated with low serum copper, absent ceruloplasmin, and high ferritin concentrations [18]. This condition may be misdiagnosed as Parkinson’s disease because of neurological symptoms [19] or HFE-related hemochromatosis because of the high ferritin concentrations and hepato-pancreatic iron accumulation[20].

##### Atransferrinemia

Atransferrinemia is characterized by the absence of transferrin that transports Fe3+ iron in the blood. Low transferrin concentration leads to microcytic anemia by deficient iron transport from the peripheral tissues (mainly the liver) to the bone marrow. Iron accumulation in organs leads to liver injury, heart failure, and splenomegaly [21].

#### Sideroblastic anemias

First, sideroblastic anemias (SA) are a group of acquired or congenital bone marrow disorders characterized by an abnormal accumulation of iron into the mitochondria of the erythroid precursors, leading to an appearance of “ring” sideroblasts on bone marrow aspiration [22].

SA can be classified as congenital or acquired. Congenital SA can be classified as syndromic or non-syndromic.

##### Non-syndromic congenital SA

The most common non-syndromic congenital SA is X-linked sideroblastic anemia (XLSA), representing around 40% of the hereditary cases [23]. It is caused by a mutation in the delta-aminolevulinic acid synthase type 2 (ALAS2) gene responsible for producing, inside the mitochondria, delta-aminolevulinic acid (ALA), which the first compound of the heme synthesis pathway [24]. It causes microcytic anemia mostly in men and older carrier women due to skewed X inactivation later in life [25].

Other causes of non-syndromic congenital SA are caused by mutation of the SLC25A38 gene coding for a transporter of the amino acid glycine into the mitochondria that ALAS2 uses for ALA synthesis. More rarely, mutations in the GLRX5 protein mitochondrial protein that plays a role in synthesizing Fe-S clusters have been described as a cause of SA [26].

##### Acquired SA

Acquired causes of SA comprise clonal myelodysplastic diseases, such as refractory anemia with ring sideroblasts appearing in late adulthood associated with other cytopenias and SF3B1 mutation. However, it primarily causes normocytic or macrocytic anemia.

Acquired SA can also be caused by copper deficiency due to malabsorption states (including gastrointestinal resections) or toxic causes such as excess zinc intake (e.g., zinc supplements, coins in some cases of pica [27]) and lead poisoning, also called saturnism [28]. Acquired SA can also be caused by the absence of functional pyridoxine (vitamin B6) needed for the function of the enzyme ALAS2. Thus, SA can be seen in cases of isoniazid treatment in tuberculosis in some patients through reduced availability of functional pyridoxine [29].

##### Syndromic congenital SA

Syndromic congenital SAs are characterized by microcytic anemia and non-hematological manifestations.

X-linked SA with ataxia is characterized by spinocerebellar ataxia in male patients that can develop as late as 45 years and is associated with anemia appearing in infancy due to a mutation in ATP-binding cassette transporter ABCB7 [30].

Congenital sideroblastic anemia-B-cell immunodeficiency-periodic fever-developmental delay syndrome (SIFD) is a form of congenital SA associated with immunodeficiency, periodic fevers, and developmental delay caused by a loss of function mutation in the TRNT1 gene coding for tRNA nucleotidyl transferase 1 involved in the maturation of cytosolic and mitochondrial transfer RNA. Some affected patients also present with hearing loss and cardiomyopathy, probably due to the energy loss induced by mitochondrial dysfunction. SIFD is characterized by recurrent fever syndrome requiring multiple hospitalizations through infancy and early childhood, immunodeficiency with B cell lymphopenia, hypogammaglobulinemia, and developmental delay. Generalized and truncal hypotonia are recurring features, often severe, progressive, and associated with gross motor developmental delay. Comprehension and communication are profoundly impaired in many children. It can be confused with a diagnosis of early onset common variable immunodeficiency [31–33].

Myopathy with exercise intolerance, lactic acidosis, SA (MLASA) syndrome, and Pearson marrow pancreas syndrome are two causes of congenital syndromic SA affecting the mitochondrial metabolism of iron. Still, they are causes of macrocytic and normocytic anemia instead of microcytic anemia [11, 12].

#### Porphyrias

Porphyrias are a group of diseases caused by mutations of genes coding for heme synthesis enzymes. Each enzymatic step can be deficient, leading to the accumulation of different heme precursors and then to various clinical symptoms [34]. Three out of the eight types of porphyrias are associated with microcytic anemia: XLSA (already developed in the section about microcytic anemia), congenital erythropoietic porphyria (CEP), and erythropoietic protoporphyria (EPP) [18].

##### CEP

CEP is caused by a mutation in the UROS gene coding for the uroporphyrinogen synthase. It leads to the accumulation of the photoreactive porphyrins uroporphyrinogen I and coproporphyrinogen I, leading to cutaneous photosensitivity reactions resulting in bullous and vesicular lesions in light-exposed areas such as the hands. Affected patients can also develop erythrodontia, osteolysis, and osteoporosis. CEP frequently manifests as hemolytic anemia in infants, but several late-onset cases in patients older than 50 have been reported [35]. Cases with microcytic anemia are linked to a mutation in the *GATA1* gene that regulates the *UROS* gene expression [36].

##### EPP

EPP is caused by a loss of function mutation of the ferrochelatase protein gene *FECH*. Ferrochelatase is a mitochondrial enzyme acting at the end of the heme biosynthesis pathway by attaching Fe2 + to protoporphyrin IX (PPIX) to produce heme. The accumulation of free PPIX is responsible for photosensitivity symptoms with burning, stinging, and pruritus after sun exposure, but notably without blistering. It is also associated with hepatic complications such as cholestasis and cytolysis. Microcytic anemia occurs in 20 to 60% of patients [37].

### Hemolytic anemia

**Fig. 2:**
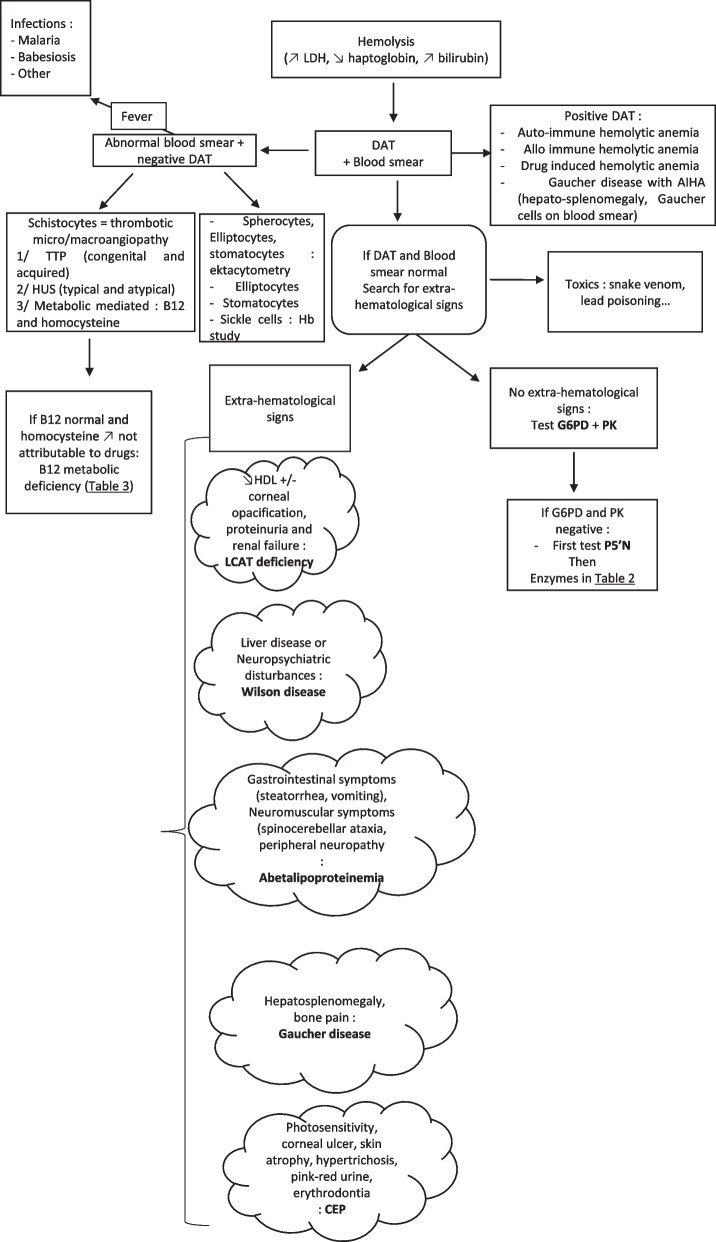
Hemolysis diagnostic pathway. CEP: congenital erythropoietic porphyria; DAT: direct antiglobulin test; G6PD: glucose-6-phosphate dehydrogenase; HDL: high-density lipoprotein, HUS: hemolytic and uremic syndrome; LCAT: Lecithin-cholesterol acyltransferase, LDH: lactate dehydrogenase; P5′N: pyrimidine 5′ nucleotidase, PK: pyruvate kinase; TTP: thrombotic thrombocytopenic purpura;

Hemolytic anemia is characterized by a reduced life span of RBCs due to increased destruction. The hallmarks of hemolytic anemia are increased reticulocyte count, unconjugated bilirubin, lactate dehydrogenase (LDH), and reduced haptoglobin. Common clinical presentations of hereditary hemolytic anemia are jaundice, splenomegaly, and recurrent abdominal pain caused by pigmented gallstone formations and blockage in the biliary tract. These signs are accompanied by signs and symptoms of anemia (dyspnea, fatigue, and conjunctival pallor) [38]. It can be caused by acquired causes like autoimmune hemolytic anemia, paroxysmal nocturnal hemoglobinuria, infectious causes (e.g., malaria, Shiga toxin-producing E. coli), or congenital causes like some types of thalassemias, membrane defects, and some IMD including RBC enzyme defects [39]. A diagnostic algorithm is proposed in Fig. 2.

#### RBC enzyme defects

RBCs are particularly vulnerable to enzymatic defects because once RBCs are produced in the blood marrow, without a nucleus, production of new enzymes isn’t possible [40].

Three enzymatic pathways essential for RBC survival can be congenitally impaired: nucleic acid metabolism, glycolysis, and glutathione generation. The diseases affecting these three pathways are summarized in Table 2**.**

**Table 2:** Enzymatic deficiencies associated with hemolytic anemia

*Most common*	
G6PD deficiency	AR
PK deficiency	AR
*Impaired mental development*	
Phosphoglycerate kinase deficiency	X-linked
Glutathione synthetase deficiency	AR
Triosephosphate isomerase deficiency	AR
*Myopathy*	
Phosphofructokinase deficiency	AR
Phosphoglycerate kinase deficiency	X-linked
*Less common without extra-hematological symptoms*	
P5′N (most common after G6PD and PK deficiency)	AR
Adenylate kinase deficiency	AR
GPI deficiency	AR
Hexokinase deficiency	AR
Glutathione reductase deficiency*	AR

AR: autosomal recessive; G6PD: glucose-6-phosphate dehydrogenase; GPI: glucose phosphate isomerase; P5′N: pyrimidine 5′ nucleotidase; PK: pyruvate kinase; *glutathione reductase deficiency can present with cataract

##### Glutathione generation impairment

RBCs produce reduced nicotinamide adenine dinucleotide phosphate and glutathione through the pentose phosphate pathway to resist oxidative stress. [40] G6PD is a crucial enzyme of this pathway. Its gene is located on the X chromosome. Thus, G6PD deficiency mainly affects male individuals. G6PD deficiency is responsible for a vulnerability to reactive oxygen species (ROS) that an extensive list of drugs, food, and infections can generate. Most affected patients are asymptomatic until they are exposed to a new source of ROS. A classic example is the ingestion of Fava beans, leading to a hemolytic crisis hour to days after the meal. It affects over 500 million individuals worldwide, with a predominance in Africa and Asia correlating with endemic areas of malaria, which G6PD deficiency offers a theoretical advantage against. However, it complicates the administration of antimalarial drugs with oxidative properties like primaquine or tafenoquine, which can lead to a hemolytic crisis. [5, 41] Diagnosis is made by testing the enzymatic activity of G6PDin RBCs. Results below 80% of the normal’s lower limit must be considered deficient. Activity can be falsely normal during a crisis or in the days following a hemolytic crisis. Thus, the best time to test for G6PD deficiency is a few weeks after the hemolytic crisis [5].

Glutathione synthetase deficiency, also known as pyroglutamic acidemia, is an IMD that usually appears in infancy. It is characterized by ataxia, hemolytic anemia, and high anion gap metabolic acidosis. Glutathione synthase is responsible for the synthesis of glutathione from gamma-glutamylcysteine. Reduced glutathione concentrations are responsible for hemolysis by vulnerability to ROS. Accumulated gamma-glutamylcysteine is converted into pyroglutamic acid, also called 5-oxo proline, responsible for metabolic acidosis. Acquired pyroglutamic acidemia can be caused by chronic or acute paracetamol ingestion, leading to glutathione depletion, the loss of negative feedback on gamma-glutamylcysteine production that causes the accumulation of gamma-glutamylcysteine converted to pyroglutamic acid. Diagnosis Is made by finding elevated levels of 5-oxoproline on a urinary organic acid panel. In suspected or confirmed cases, paracetamol is contraindicated [42, 43].

Glutathione reductase deficiency is responsible for the lack of available reduced glutathione. It is associated with hemolytic anemia and early-onset cataract. Hemolytic crises are caused by the same sources of oxidative stress as in G6PD deficiency. It is diagnosed by measuring the reduced activity of glutathione reductase in RBCs. Glutathione reductase activity needs flavin-adenine dinucleotide (FAD) as a cofactor. FAD is derived from Riboflavin (vitamin B2). B2 deficiency from poor dietary intake can thus be a cause of acquired glutathione reductase deficiency [44].

##### Glycolysis impairment

ATP production needed by the RBCs to survive relies on anaerobic glycolysis because of the absence of mitochondria. Pyruvate kinase (PK) is a mainstay enzyme of this pathway, and its deficiency is the most common cause of hemolytic anemia due to impaired glycolysis. Diagnosis is based on reduced PK activity and *PLKR* gene testing. Glucose phosphate isomerase deficiency is the second most common enzymopathy of glycolysis. Neuromuscular symptoms and intellectual disability may be present. The other less common deficiencies of glycolysis are triosephosphate isomerase deficiency, Hexokinase deficiency, Phosphofructokinase deficiency, and Phosphoglycerate kinase deficiency [39].

##### Nucleic acid metabolism impairment

The most frequent impairment in nucleic acid metabolism leading to hemolytic anemia is pyrimidine 5′ nucleotidase (P5′N) deficiency. In this autosomal recessive genetic disorder, unmetabolized pyrimidine nucleotides accumulate in the RBC and precipitate, forming basophilic stippling visible on blood films. The diagnosis relies upon demonstrating high concentrations of pyrimidine nucleotides and a reduced P5′N-1 activity in RBC. In a review of 64 patients reported in the literature, the age of diagnosis ranges from 3 months to 64 years, with a median of 15 years. [45, 46] A less common cause of hemolytic anemia impaired by nucleic acid metabolism is adenylate kinase deficiency associated with psychomotor impairment in some cases [47].

#### Cytotoxic anemia

Hemolytic anemia can also result in the accumulation of cytotoxic metabolic products in IMDs. The most frequent ones are Wilson disease and porphyria.

##### Wilson disease

Wilson disease is an autosomal recessive genetic disease caused by a mutation in the transmembrane copper transporter ATP7B. In physiological conditions, copper enters hepatocytes through a copper transporter CTR1. It is then attached to metallothionein and can be incorporated into apo ceruloplasmin to make ceruloplasmin enter the Golgi apparatus using the ATP7B transporter. In Wilson disease, the absent ATP7B transporter leads to the accumulation of copper inside the liver and the release of free copper, which is not incorporated into ceruloplasmin. Free copper can cause neurological tissue damage with astrogliosis, demyelination, tissue necrosis, or liver tissue damage with chronic hepatitis and fibrosis. Wilson’s disease clinical presentation varies widely. The standard features are liver disease leading to cirrhosis, neuropsychiatric disturbances ranging from atypical schizophrenia to parkinsonism, and Kayser-Fleischer rings. Hemolytic anemia is a crucial presenting feature as it can be the first sign of Wilson disease before the neurological or the hepatic manifestations. There should be high clinical suspicion for hemolytic anemia in children and young adults as an early treatment with chelating agents can prevent the development of severe liver disease or neurological deficit [48].

If Wilson disease is suspected, four copper tests should be performed: serum ceruloplasmin dosage, exchangeable copper (CuEXC) evaluation, 24-h urinary copper, and serum copper. In typical cases, serum ceruloplasmin is decreased, urinary copper levels are increased, serum copper is decreased, and CuEXC is increased. It is possible to calculate a ratio of CuEXC and total serum copper called relative exchangeable copper (REC), which allows to diagnose Wilson's disease with a sensitivity and specificity of nearly 100% when using a cut-off value of > 18.5%. Confirmation can be made through molecular biology study of the *ATP7B* gene [49–51].

##### Porphyria

Porphyrias were already discussed in the microcytic anemia section. Still, CEP can also cause hemolytic anemia. In a review of 128 cases of CEP from 1874 to 1994 of patients with ages of onset ranging between birth and 63 years old, 60% had hemolytic anemia [52].

#### Thrombotic microangiopathy

Hemolytic anemia can also be associated with thrombopenia and schistocytes on blood smears in thrombotic microangiopathy (TMA). Classical thrombotic microangiopathies are acquired or congenital thrombotic thrombocytic purpura and hemolytic and uremic syndrome. Vitamin B12 (cobalamin) deficiencies or defective cobalamin metabolism can mimic TMA with low platelets and fragmented RBCs associated with organ damage. This condition, sometimes called metabolic-mediated TMA, should be considered in the differential diagnosis of TMA. Signs of cobalamin deficiency or defective metabolism are a low reticulocyte count in the presence of elevated LDH and macrocytosis. Diagnostic suspicion is consolidated by high homocysteine or low vitamin B12 levels.

One of the most frequent cobalamin metabolism deficiencies is a deficiency in the *MMACHC* gene (CblC) coding for Methylmalonic aciduria and homocystinuria type C protein that helps convert cobalamin into adenosylcobalamin and methylcobalamin. Accumulation of homocysteine is thought to play a role in the pathogenesis of TMA by renal vascular endothelial toxicity associated with small vessel injury caused by hypomethionemia [53–55].

The chapter about macrocytic anemia below further develops the etiology of congenital cobalamin metabolism defects and cobalamin deficiency.

#### Gaucher disease

Gaucher disease is an IMD characterized by the buildup of glucocerebrosides in macrophages caused by glucocerebrosidase deficiency. After the buildup of glucocerebrosides, the macrophages infiltrate the liver, the bone marrow, and the spleen, leading to hepatosplenomegaly, cytopenia, and bone pain. It can rarely be associated with hemolytic anemia. Anemia is partly caused by hypersplenism, but abnormal RBC phagocytosis has been reported to be linked to removing dysmorphic erythrocytes. In untreated patients with Gaucher disease, various abnormal RBC shapes have been reported, including schistocytes, dacryocytes, and echinocytes. [56] Auto-immune hemolytic anemia is also more common in patients with Gaucher disease than in the general population, with reported incidences varying between 0.55 and 2.7% [57].

#### Lipid metabolism

The RBC membrane is composed of a regulated amount of lipids, including cholesterol, phosphatidylcholine, phosphatidylethanolamine, sphingomyelin, and ceramide. [58] Enzymatic deficiencies in lipid metabolism pathways can influence cholesterol membrane constitution and be associated with hemolysis.

##### Lecithin-cholesterol acyltransferase (LCAT) deficiency

LCAT is an enzyme involved in the synthesis of HDL cholesterol. LCAT deficiency is an autosomal receive disease characterized by low HDL levels, and depending on the mutation of the LCAT gene, it can lead to premature corneal opacification, hemolytic anemia, proteinuria, and renal failure [59, 60].

##### Abetalipoproteinemia

Abetalipoproteinemia is characterized by the absence of apolipoprotein B. It is caused by a microsomal triglyceride transfer protein (MTTP) deficiency. Without MTTP, lipids can’t be assembled to form Chylomicrons in the intestines and VLDL in the liver, leading to the accumulation of lipids in intestinal epithelial cells and hypolipidemia with a deficiency of fat-soluble vitamins. Clinical manifestations include hematological signs with acanthocytosis and hemolysis. There are neuromuscular symptoms resulting from vitamin E malabsorption, including spinocerebellar ataxia, peripheral neuropathy, and myopathy, with the loss of deep tendon reflex described as an early sign of neurological involvement. There are also gastrointestinal symptoms such as vomiting, chronic diarrhea with steatorrhea, and failure to thrive. An endoscopic examination can find a snowy white appearance of the small intestine resulting from the accumulation of lipids, also called a snow-white duodenum [61].

### Megaloblastic anemia

Megaloblastic anemia is a type of macrocytic anemia associated with vitamin B12 (cobalamin) or folate deficiencies. It is caused by impaired DNA synthesis by reduced availability of cobalamin or folates caused by poor oral intake, malabsorption, drugs, or IMDs. Diagnosis is made by examination of a peripheral blood smear or bone marrow film. Findings include RBCs with mean corpuscular volume (MCV) > 100 fl but sometimes > 120 fl, macroovolocytes, hypersegmented neutrophils, and the presence of other cytopenias.

The first diagnostic step involves ruling out hemolysis, which can increase MCV through increased reticulocytosis. Then, a dosage of folates, vitamin B12, and TSH is used to rule out vitaminic deficiencies and hypothyroidism. The next step is usually a bone marrow aspiration to look for signs of myelodysplasia. We underline the need to realize a dosage of methylmalonic acid and homocysteine if folates and vitamin B12 are normal, especially in younger patients, to look for impairments in cobalamin and folate deficiencies that are developed underneath. If methylmalonic acid and homocysteine are normal without signs of myelodysplasia, megaloblastic anemia can be caused by thiamine-responsive megaloblastic anemia.

**Fig. 3:**
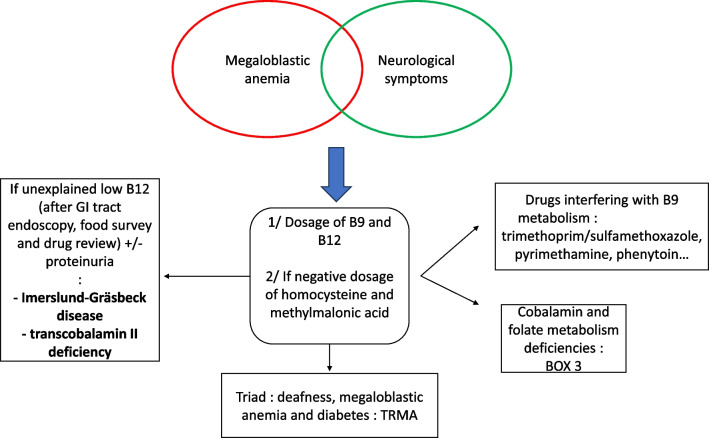
Macrocytic anemia. GI gastrointestinal, TRMA: thiamine-responsive megaloblastic anemia

A diagnostic algorithm is proposed in Fig. 3**,** and IMD associated with megaloblastic anemia are summarized in Table 3.

**Table 3:** IMDs associated with megaloblastic anemia

	Clinical signs	Biological signs	Gene (protein)	Specific biological sign
*Impaired cobalamin absorption*				
Imerslund-Gräsbeck	Impaired absorption of B12	Proteinuria Low plasmatic cobalamin	*CUBN* (cubulin) or *AMN* (amnionless)	↘ plasmatic cobalamin
Transcobalamin II deficiency			*TCN2* (transcobalamin 2)	↘ plasmatic cobalamin
*Impaired cobalamin metabolism*				
MMA	Developmental delay, progressive renal failure (tubulointerstitial nephritis), metabolic strokes, optic nerve atrophy	Metabolic acidosis, ketosis, hypoglycemia, hyperammonemia, neutropenia		Urinary organic acids: ↗ methylmalonic acid ↗ methylcitrate
MUT deficiency			*MUT* (methylmalonyl-CoA mutase)	
cblA type			*MMAA* (MMA type A protein)	
cblB type			*MMAB* (cobalamin adenosyltransferase)	
cblC type			*MMACHC* (MMA and homocystinuria type C protein)	
cblD type			*MMADHC* (MMA and homocystinuria type D protein)	
cblF type			*LMRBD1* (Lysosomal cobalamin transport escort protein LMBD1)	
PA	Same as MMA + cardiomyopathy (9–23% of patients)	Same as MMA	*PCCA* (Propionyl-CoA Carboxylase Subunit Alpha) or *PCCB* (Propionyl-CoA Carboxylase Subunit Bêta)	Urinary organic acids: N methylmalonic acid↗ methylcitrate
Methionine synthase reductase deficiency (cblE type)	Severe neonatal form: seizures, encephalopathy, hypotonia Adult attenuated form: psychiatric symptoms, acute neurological changes		*MTRR* (Methionine synthase reductase)	↗ homocysteinemia and ↘ methioninemia
Methionine synthase deficiency (cblG)			*MTR* (5-Methyltetrahydrofolate-Homocysteine Methyltransferase)	
*Impaired folate metabolism*				
MTHFD1 deficiency	Severe combined immunodeficiency with infections (repeated or opportunistic) and autoimmunity		*MTHFD1* (methylenetetrahydrofolate dehydrogenase 1)	↗homocysteine N methylmalonic acid, N methionine, N cobalamin, N folate
DHFR deficiency			*DHFR* (dihydrofolate reductase)	N total serum folate, N homocysteine, ↘ specific red blood cell folate levels ↘ CSF 5-methyl-tetrahydrofolate
Formiminotransferase-cyclodeaminase deficiency			*FTCD* (Formiminotransferase-cyclodeaminase)	↗ plasma and urine formiminoglutamic acid
*Impaired B1 metabolism*				
TRMA	Triad: megaloblastic anemia improving with B1 supplementation, diabetes mellitus, and deafness		*SCLC19A2* (SCLC19A2)	

CSF: cerebrospinal fluid; DHFR: Dihydrofolate reductase; IMD: inherited metabolic diseases; MMA: methylmalonic acidemias; MTHFD1: methylenetetrahydrofolate dehydrogenase; N: normal; PA: propionic acidemia; TRMA: thiamin responsive megaloblastic anemia; ↗: increased;↘: decreased

#### Impaired cobalamin metabolism

Cobalamin is a coenzyme and cofactor used for DNA synthesis, methionine synthesis from homocysteine, and conversion of propionyl into succinyl coenzyme A from methylmalonate [62].

Lack of available active forms of cobalamin leads to hematological manifestations ranging from isolated macrocytosis to pancytopenia due to decreased DNA synthesis. Neuropsychiatric symptoms include sensitive polyneuritis, combined sclerosis of the spinal cord, and dementia caused by impaired myelin sheath production using the methylmalonate pathway. It is also associated with atherosclerotic disease linked to hyperhomocysteinemia [63].

Cobalamin is found naturally in meat and dairy products. Its deficiency can be caused by poor intake in vegetarian but mostly vegan patients. It can be prevented with B12 supplements [64]. Cobalamin deficiency can be caused by malabsorption due to a defect in one of the steps leading to cobalamin absorption. The first step is the dissociation of cobalamin from food proteins that need gastric acid production. Thus, atrophic gastritis, partial gastrectomy, and chronic PPI use reduce cobalamin absorption. The second step is attachment to intrinsic factor (IF), whose production is impaired in pernicious anemia, but intrinsic factor gene loss of function mutations have also been described [65]. The third step is absorption through the cobalamin-IF receptor on the ileal mucosa [63]. This receptor comprises two subunits: cubulin and amnionless. Imerslund-Gräsbeck syndrome is caused by a mutation of one of these subunits. It manifests as selective malabsorption of cobalamin discovered between a few months or 15 years of age, frequently associated with mild proteinuria linked to the role of this receptor in renal protein reabsorption pathways. More regularly, ileal absorption is impaired by ileal resections and infectious or inflammatory lesions [62, 66].

After intestinal absorption, cobalamin entry and transport in the blood circulation is mediated by the protein transcobalamin II. This protein can be congenitally deficient and responsible for severe cobalamin deficiency symptoms early in infancy [67].

Methylmalonic acidemias (MMA) are a group of IMDs resulting from a failure to convert methylmalonyl-coenzyme A (CoA) into succinyl-CoA used by the citric acid cycle. It is caused by deficient activity of methyl malonyl-CoA mutase (MUT). It is classified by the cause of the reduced activity of MUT: MUT deficiency or a deficiency in the metabolism of cobalamin, a MUT coenzyme.

Causes of a defect in cobalamin metabolism are anomalies of uptake of cobalamin from the lysosome by LMBRD1 deficiency (cblF), intracellular transport deficiency by *MMACHC* or *MMADHC* mutations (cblC and cblD, respectively), impaired synthesis of functional 5′-deoxy-adénosylcobalamine by *MMAA* (cblA) or *MMAB* (cblB) mutations.

The onset of manifestations in MMA ranges from the neonatal period to adulthood. Besides macrocytic anemia, the main signs are metabolic acidosis, ketosis, hypoglycemia, hyperammonemia, and neutropenia. It is associated with developmental delay, progressive renal failure from tubulointerstitial nephritis, metabolic stroke, and optic nerve atrophy. Diagnosis is supported by finding a high blood homocysteine levels and high concentration of methylmalonic acid and methylcitrate on an organic acids urine test [68, 69].

Propionic acidemia (PA) is another type of organic aciduria caused by a mutation in the propionyl-Coa reductase impairing the conversion of Propionyl-CoA into Methylmalonyl-Coa, the first step of the pathway leading to succinyl-Coa and the citric acid cycle. As in MMA, organic acids such as methylcitrate can be increased, but not methylmalonic acid. Symptoms are similar to MMA but with an increased proportion of described cardiomyopathy: 9–23% of cases for PA compared to a few cases reported in MMA [68, 70].

Methionine synthase reductase (cblE) and methionine synthase (cblG) defects are inborn errors of cobalamin metabolism, leading to impairment of the remethylation of homocysteine to methionine. In severe forms, patients present in the neonatal period with seizures, encephalopathy, macrocytic anemia, and hypotonia. In attenuated patients, symptoms may present in adulthood with acute neurological changes or psychiatric symptoms, with first symptoms appearing as late as 20 years, so as in most cobalamin metabolism deficiencies, the central and peripheral nervous system and the bone marrow are affected. Biological diagnosis relies on the association of hyperhomocysteinemia and hypomethioninemia [71, 72].

#### Impaired folate metabolism

Methylenetetrahydrofolate dehydrogenase 1 (MTHFD1) functions in the cytoplasmic folate cycle, where it is involved in de novo purine synthesis, synthesis of thymidylate, and remethylation of homocysteine to methionine. MTFHD1 deficiency can be responsible for severe combined immunodeficiency and is associated with an atypical hemolytic uremic syndrome, infections, and autoimmunity. Homocysteine levels are increased, but methylmalonic acid, methionine, cobalamin, and folate levels are normal. Suspicion should be raised in case of macrocytic anemia appearing in early childhood associated with opportunistic or repeated infections [71, 73].

Common causes of folate deficiency are poor oral intake (of green leafy vegetables, legumes, and cereal products), alcoholism, and poor intestinal absorption. Some medications can impair folate metabolism, including methotrexate, trimethoprim/sulfamethoxazole, pyrimethamine, and anticonvulsants such as phenytoin [74].

IMDs leading to megaloblastic anemia through impaired folate metabolism are caused by enzyme deficits leading to DNA synthesis: Dihydrofolate reductase (DHFR) deficiency and formiminotransferase-cyclodeaminase deficiency.

DHFR catalyzes the reduction of dihydrofolate to tetrahydrofolate and, at a lower rate, of folic acid to DHF. DHFR deficiency caused by homozygous mutations causes megaloblastic anemia and cerebral folate deficiency, responsible for neurological symptoms responsive to folinic acid administration. Total serum folate and homocysteine levels are normal, but specific red blood cell folate levels are low, and cerebrospinal fluid analysis reveals low 5-methyl-tetrahydrofolate [75, 76].

Formiminotransferase-cyclodeaminase deficiency is responsible for formiminoglutamic aciduria, an autosomal recessive disorder of histidine and folate metabolism. It is characterized by elevated plasma and urine formiminoglutamic acid. Severe forms are associated with developmental delay and anemia [77, 78].

#### Thiamine-responsive megaloblastic anemia (TRMA)

TRMA is an IMD caused by a mutation of SLC19A2, a thiamine (vitamin B1) transporter. SCLC19A2 is mainly expressed in hematopoietic stem cells, pancreatic islet cells, and the inner ear. As a result, the classical triad of TRMA is megaloblastic anemia, diabetes mellitus, and deafness. TRMA has also be linked with ophthalmologic manifestations such as optic nerve atrophy, and retinitis pigmentosa. Thiamine is a cofactor of transketolase, an enzyme of the pentose cycle needed for ribose synthesis. Macrocytic anemia in TRMA is linked to impaired DNA synthesis through the loss of ribose synthesis. TRMA is also caused by inhibiting the ALAS2 enzyme, responsible for a sideroblastic pattern on blood marrow aspirations. Diagnosis is made by genetic testing of *SLC19A2* [79, 80].

## Thrombocytopenia

Thrombocytopenia is a low count of platelets caused by decreased medullary production, increased consumption in clot formation, increased destruction, or splenic sequestration in splenomegaly.

Cobalamin and folate metabolism deficiencies are responsible for thrombocytopenia associated with macrocytic anemia and neutropenia.

IMD can affect all the different mechanisms of thrombocytopenia.

### Sitosterolemia

Sitosterolemia is characterized by the accumulation of phytosterols in blood and tissues. Clinical features comprise xanthomas, premature atherosclerosis, arthralgia, splenomegaly with elevated total cholesterol, low-density lipoprotein cholesterol, and plasma phytosterol levels.

It can manifest with macrothrombocytopenia and anemia associated with stomatocytes. In a case series of 5 adults from 24 to 63 years at diagnosis, the initial symptoms were anemia in 2 patients and thrombocytopenia in one patient.

It is caused by a mutation in *ABCG5* and *ABCG8* genes coding for ATP transporters localized on the apical border of enterocytes. ABCG5 and ABCG8 are responsible for active sterol excretion.

Diagnosis is supported by finding elevated levels of plasma phytosterols, sitosterol, and campesterol and confirmed with genetic testing of *ABCG5* and *ABCG8* [81, 82].

### Galactose epimerase deficiency

Galactose epimerase (GALE) deficiency is a congenital disorder of glycosylation. It can lead to severe thrombocytopenia characterized by dysplastic megakaryocytes. More rarely, it can be associated with anemia and febrile neutropenia. It has many clinical symptoms in infants, including hypotonia, poor feeding, vomiting, hepatomegaly, and cataracts. Diagnosis is made by measuring GALE activity in RBCs [83, 84].

### Gaucher disease

In a multicenter study in Italy, Gaucher disease was associated with thrombocytopenia in 10/15 patients, and one had isolated thrombocytopenia [85].

### Acid sphingomyelinase deficiency (ASMD)

ASMD, formerly known as Niemann pick disease, is a lysosomal storage disease caused by a deficiency in sphingomyelinase activity. This deficiency leads to the accumulation of sphingomyelin and other lipids within tissues rich in reticuloendothelial cells, including the spleen, liver, lung, bone marrow, and lymph nodes. Type B ASMD has a benign prognosis in contrast with type A ASMD involving severe neurological disease in infancy. ASMD type B can be associated with thrombocytopenia. Cases often involve hypersplenism with splenomegaly and pancytopenia, but cases presenting as isolated thrombocytopenia with mild splenomegaly have been described in patients as old as 57 sears. Diagnosis is made by analysis of sphingomyelinase activity below normal levels in leukocytes and fibroblasts [86].

## Neutropenia

Neutropenia is a reduced number of circulating neutrophils in the blood caused by excess margination, decreased medullary production, or splenic sequestration. The risk of neutropenia is a susceptibility to infections.

### Type Ib glycogen storage disease

Glycogen storage diseases (GSD) are a group of inborn disorders of glycogen metabolism. Glycogen is a polysaccharide form of glucose that can be stored in muscles and the liver as a source of energy. GSD Ib is caused by Glucose-6-phosphate (G6P) translocase deficiency. Common symptoms are hepatomegaly and severe hypoglycemia. Neutrophils have a dysfunctional metabolism caused by the accumulation in the cytosol of a structural analog of G6P called 1,5-anhydroglucitol-6-phosphate that is also transported by G6P translocase from the cytosol into the endoplasmic reticulum. Accumulation of this toxic metabolite inhibits glycolysis. The lack of available glycolysis deprives the neutrophils of their sole energy source, leading to apoptosis and neutropenia and defective bactericidal activity, leading to infections, ano-urogenital lesions, and inflammatory bowel disease [87].

### Barth syndrome

Barth syndrome is a congenital X-linked disorder of lipid metabolism. It is caused by a mutation of Tafazzin, a protein involved in phospholipid synthesis. Tafazzin mutation leads to a deficient cardiolipin synthesis. It is characterized by muscle weakness, growth retardation, dilated cardiomyopathy, and 3-methylglutaconic aciduria. It is associated with variable neutropenia. Elevated blood and urine 3-methylglutaconic acid, a marker of aberrant aerobic energy metabolism, raises suspicion for Barth syndrome. Diagnosis is made by identification of a high ratio of monolysocardiolipin to cardiolipin or genetic testing of the *TAZ* gene [88, 89].

### Shwachman-diamond syndrome (SDS)

SDS is an inherited ribosomopathy caused by mutations in the SBDS, DNAJC21, or EFL1 genes coding for proteins involved in the maturation and stabilization of ribosomal subunits.

It is characterized by exocrine pancreatic deficiency, bone marrow failure, and predisposition to myeloid leukemia. One of the most frequent signs of SDS is neutropenia, affecting between 88 and 100% of patients. It can also cause anemia in 42–82% of cases, thrombocytopenia reported in 24%–88% of cases, or pancytopenia in 10–65% of patients with some developing aplastic anemia [89].

## Pancytopenia

Pancytopenia is defined as a decrease in the three bloodlines: thrombocytopenia, anemia, and leukopenia. The causes of pancytopenia are extensive and cannot all be detailed in this review, but a diagnostic approach to pancytopenia has been proposed by Gnanaraj et al. [90].

Two mechanisms are involved in pancytopenia: impaired medullary production or peripheral destruction. Some IMD can cause pancytopenia through one or two of these mechanisms, with impaired peripheral destruction mainly represented by situations of hypersplenism.

### Impaired medullary production

In IMD, medullary production can be decreased in situations of impaired metabolism of cobalamin and folates (developed in the chapter about megaloblastic anemia) or the accumulation of toxic metabolites impacting the development of hematological stem cells.

#### Organic acidemias (OAD)

Organic acidemias (OAD) are a group of IMD characterized by the accumulation of non-amino acids called “organic acids,” which can be detected in urine, plasma, and cerebrospinal fluid. This accumulation is caused by deficient mitochondrial breakdown of CoA-activated carbonic acids such as propionyl-CoA,methylmalonyl-CoA, isovaleryl-CoA, and glutaryl-CoA. Some of the resulting organic acids are thought to bear a direct toxicity to mitochondria, which leads to dysfunction of high energy-demanding organs such as the heart, the optic nerve, the liver, the brain, and the hematological precursor cells.

The three most common OAD are propionic acidemia, isovaleric acidemia, and methylmalonic acidemia, which has already been discussed in the section about macrocytic anemia. Metabolic decompensations in OAD can be triggered by infections or excessive protein intake. They should thus be on the list of differential diagnoses in patients presenting with recurring unexplained pancytopenia in the context of fever. OAD should also be considered a differential diagnosis in infections and high anion gap metabolic acidosis not entirely explained by renal failure or lactic acidosis [91, 92].

Diagnostic workup of these diagnoses involves measuring methylmalonic acid levels raised during acute illness, followed by a urine organic acid profile and genetic testing (Table 3).

#### Lysinuric protein intolerance (LPI)

LPI is caused by a homozygous loss of function mutation in the *SLC7A7* gene coding for a cationic amino acid transporter subunit called y + L-type amino acid transporter 1 (y + LAT1). This cationic amino acid transporter is found mainly in intestinal and renal cells, but y + LAT1 is also expressed in the lungs, spleen, circulation lymphocytes, and macrophages. This transporter's primary function is the reabsorption of three dibasic amino acids: arginine, ornithine, and lysine.

LPI is associated with a broad spectrum of symptoms, namely digestive symptoms (vomiting, diarrhea, hepatosplenomegaly, and aversion to protein-rich foods), neurological developmental delay, renal tubulopathy possibly leading to renal failure, proteinuria, and pulmonary alveolar proteinosis. Cytopenias are found in 37% of LPI cases, mainly normocytic anemia and thrombopenia, but neutropenia and pancytopenia have been reported. The mechanisms evoked for cytopenia in LPI are a hemophagocytic process in the bone marrow and a hypersplenism with a prevalence of splenomegaly up to 62%.

When LPI is suspected, amino acid levels should be measured in the urine, showing high arginine, ornithine, and lysine levels compared with normal urinary cystine levels. Diagnosis is confirmed by genetic testing of the *SLC7A7* gene [93, 94].

### Hypersplenism

Hypersplenism can be defined as cytopenia induced by splenomegaly. The mechanisms involve retention of cells in the spleen, phagocytosis, and autoimmunity. All causes of splenomegaly can induce cytopenia by hypersplenism.

Splenomegaly can be caused by a variety of different diseases, including hematological conditions (e.g., chronic myelocytic leukemia, chronic lymphocytic leukemia, polycythemia vera, gray platelet syndrome, chronic hemolysis), portal hypertension (due to cirrhosis or abdominal veinous thrombosis), Systemic diseases (e.g., sarcoidosis, systemic lupus erythematosus, rheumatoid arthritis with sometimes a Felty syndrome), infections (e.g. endocarditis, infectious mononucleosis, leishmaniasis) and IMD.

The group of IMD that are often linked to splenomegaly are storage diseases. These diseases are characterized by the accumulation of enzymatic substrates in different organs. In the spleen, this accumulation leads to splenomegaly. Storage diseases strongly associated with splenomegaly include Gaucher disease, mucopolysaccharidoses, acid sphingomyelinase deficiency, Niemann-Pick type C, LCAT, Tangier disease, and cholesterol ester storage disease.

The diagnostic work-up of splenomegaly is extensive and goes beyond the scope of this review, but splenomegaly, hypersplenism, and especially in the context of heredity have been the subject of a narrative review by Weinreb et al. [95, 96].

## Blood smear and suggestive clinical situations

Thus, in addition to vitamin deficiencies, iron deficiencies, and other classical causes that can cause cytopenia in IMD, two different situations are usually encountered in clinical practice: 1) Cytopenia as the primary manifestation of IMD, such as hemolytic anemia in G6PD deficiency; 2) Cytopenia as one of many manifestations in a more complex clinical picture, such as in Gaucher disease.

Some clinical situations highly suggest certain IMD; a subjective top 5 of these “open and shut” cases have been highlighted in Table 4 based on our clinical experience.

**Table 4:** Top 5 “open and shut” clinical situations

Diagnosis to recall	Clinical situations
G6PD deficiency	Favism (ingestion of fava beans and hemolysis), Rasburicase induced hemolysis
Gaucher disease	Hypersplenism and Ashkenazi Jewish decent (1/450 births in this population) [8]
IRIDA syndrome	Iron deficiency in young patients without an etiology and not responding to iron supplementation
Porphyria	Cutaneous manifestations and anemia (search for darkening urine after light exposition)
Wilson disease	Hemolytic anemia with hepatic cytolysis or Parkinsonism,

G6PD: glucose-6-phosphate dehydrogenase, IRIDA: Iron refractory iron deficiency anemia

Blood smear can be a useful diagnostical tool; an additional list of “IMD linked with blood smear abnormalities” is provided in Additional file 1.

**Fig. 4:**
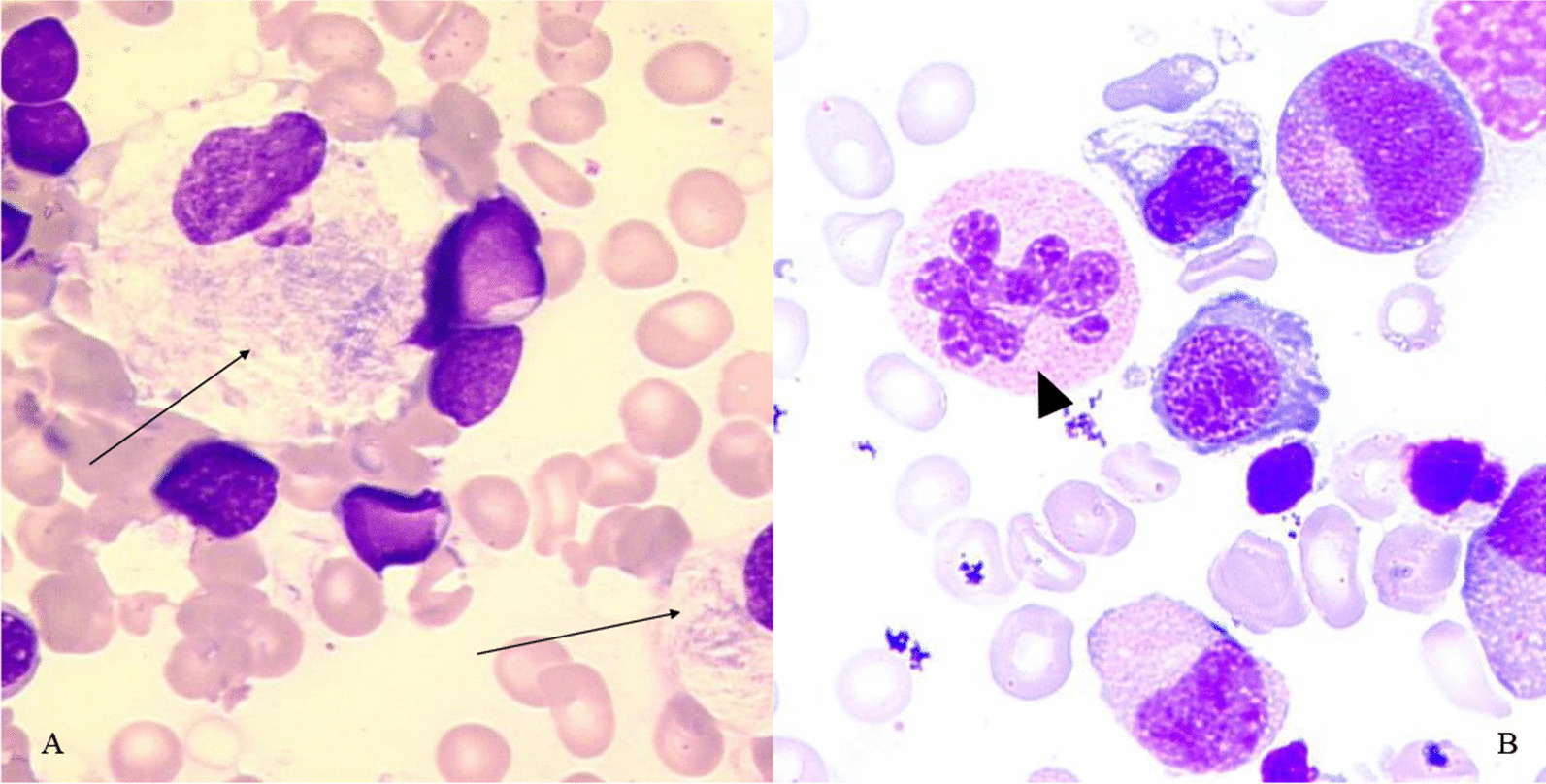
A. May-Grünwald Giemsa (MGG) stained blood smear at high magnification (× 100) showing two Gaucher cells with typical crumpled, fibrillary cytoplasm with a bluish-gray tinge. B. MGG stained blood smear at high magnification (× 100) in a case of megaloblastic anemia, showing one hypersegmented neutrophil

In Gaucher disease, pathological macrophages can be found in the bone marrow or on a peripheral blood smear called “Gaucher cells” (Fig. 4A). Gaucher cells are large with a small nucleus inside a cytoplasm with a fibrillar appearance, reminding wrinkled tissue paper. This fibrillary appearance is due to an elongation of lysozymes by the accumulation of glucocerebrosides [97].

In macrocytic anemia, hypersegmented neutrophils, which visually possess six or more segments, are a clue for megaloblastic anemia [98] (Fig. 4B). In G6PD deficiency, blood smear can be normal but can also show suggestive anomalies: bite cells, hemighost cells, and Heinz bodies on supravital staining [5]. Stomatocytes can be a clue for the diagnosis of sitosterolemia [81].

## Conclusion

IMD are rare genetic diseases. Diagnosis is often made during infancy or early childhood, but atypical clinical presentations, mild symptoms, or later onset of symptoms can lead to a diagnosis made during adulthood. Physicians taking part in diagnosing cytopenia in adults should be aware of IMD, which can present with only hematological signs or be accompanied by hematological signs during their evolution. These physicians should have a high degree of suspicion for IMD in case of cytopenia arising during childhood but also in cases without a definite diagnosis, not responding to usual treatment, or with signs of involvement of other organs.

The identification of IMD can often lead to a specific treatment due to the progress of the treatment of IMD in recent years.

### Supplementary Information

**Additional file 1.** IMD linked with blood smear abnormalities.

## Data Availability

No new data were created or analysed during this study. Data sharing is not applicable to this article.

## Abbreviations

ALA: Delta-aminolevulinic acid
ALAS2: Delta-aminolevulinic acid synthase type 2
ASMD: Acid sphingomyelinase deficiency
CEP: Congenital erythropoietic porphyria
CoA: Coenzyme A
CRP: C-reactive protein
CSF: Cerebrospinal fluid
CuEXC: Exchangeable copper
DAT: Direct antiglobulin test
DHFR: Dihydrofolate reductase
DMT1: Divalent metal transporter 1
EPP: Erythropoietic protoporphyria
FAD: Flavin-adenine dinucleotide
G6PD: Glucose-6-phosphate dehydrogenase
G6P: Glucose-6-phosphate
GALE: Galactose epimerase
GI: Gastrointestinal
GSD: Glycogen storage diseases
Hb: Hemoglobin
HDL: High-density lipoprotein
HPLC: High-performance liquid chromatography
HUS: Hemolytic and uremic syndrome
IF: Intrinsic factor
IMD: Inherited metabolic disease
IRIDA: Iron refractory iron deficiency anemia
IV: Intravenous
LCAT: Lecithin-cholesterol acyltransferase
LDH: Lactate dehydrogenase
MCV: Mean corpuscular volume
MLASA: Myopathy with exercise intolerance, lactic acidosis, and sideroblastic anemia
MMA: Methylmalonic acidemias
MTFHD: Methylenetetrahydrofolate dehydrogenase
MTTP: Microsomal triglyceride transfer protein
MUT: Methyl malonyl-CoA mutase
NPD: Niemann-Pick disease
OAD: Organic acidemias
P5′N: Pyrimidine 5′ nucleotidase
PA: Propionic acidemia
PK: Pyruvate kinase
PPI: Proton pump inhibitor
PPIX: Protoporphyrin IX
RBC: Red blood cells
REC: Relative exchangeable copper
ROS: Reactive oxygen species
SA: Sideroblastic anemia
SDS: Shwachman-Diamond syndrome
SIFD: Congenital sideroblastic anemia-B-cell immunodeficiency-periodic fever-developmental delay syndrome
TIBC: Total iron binding capacity
TMA: Thrombotic microangiopathy
TRMA: Thiamine-responsive megaloblastic anemia
XLSA: X-linked sideroblastic anemia
XLSA-A: X-linked sideroblastic anemia with ataxia
y + LAT1: Y + L-type amino acid transporter 1
