# Supplementary material for: Blood cytopenias as manifestations of inherited metabolic diseases: a narrative review
Source: Orphanet J Rare Dis. 2024 Feb 14;19:65. doi: 10.1186/s13023-024-03074-4 (PMC10865644; doi:10.1186/s13023-024-03074-4)
Supplement: Supplementary file 1 — Additional file 1. IMD linked with blood smear abnormalities. [file 13023_2024_3074_MOESM1_ESM.pdf]

Peripheral blood smear abnormalities in IMD (adapted from Crushell E, Clarke JTR, Nyhan WL. Hematological disorders. In : Zschocke J, Hoffmann GF. Inherited metabolic diseases: A Clinical Approach. Springer; 2010. p. 233-241.)

Cell type	Morphology	Disorder
Red cells	Target cells	Lecithin: cholesterol acyltransferase deficiency<br>Abetalipoproteinemia<br>Sideroblastic anemia
	Spherocytes	Hypersplenism<br>G6PD deficiency<br>Pyruvate kinase deficiency
	Spiculated red cells (acanthocytes or echynocytes)	Wolman disease<br>Cbl C defects<br>Hallervorden-Spatz (panthothenate kinase) syndrome<br>Pyruvate kinase deficiency<br>Phosphoglycerate kinase deficiency<br>Aldolase deficiency<br>Abetalipoproteinaemia
	Heinz body	G6PD deficiency<br>Wilson disease<br>Methemoglobinemia
	Howell-Jolly body	Megaloblastic anemia
	Macrocytosis	Megaloblastic anemia (vitamin B12 or folate deficiency)
	Megalocytes (oval shape)	Megaloblastic anemia
	Pappenheimer body (siderocyte)	Sideroblastic anemia
	Crystals	Erythropoietic porphyria
	Irregularly contracted cells	G6PD deficiency (blister cells)<br>Wilson disease<br>Glutathione biosynthesis deficiency<br>Glutathione peroxidase deficiency
White cells	Basophilic stippling	Pyrimidine-5’ nucleosidase deficiency
	Vacuolated lymphocytes	Aspartylglucosaminuria<br>Multiple sulfatase deficiency<br>Mucolipidosis II<br>GM_1 gangliosidosis<br>Mucopolysaccharidosis<br>Niemann-Pick disease Type A, C<br>Pompe disease<br>Sialidosis<br>Wolman’s disease<br>Salla disease<br>Batten’s disease (neuronal ceroid lipofuscinosis)<br>Fucosidosis<br>Mannosidosis
	Vacuolated leucocytes (Jordan’s anomaly)	Dorfman-Chanarin syndrome<br>Wolman syndrome<br>Carnitine deficiency
	(Alder) Reilly bodies	Mucopolysaccharidoses
	“Sea blue” histiocytes	Niemann-Pick disease<br>Ceroid lipofuscinoses<br>Adult cholesterol ester storage disease<br>GM_1 gangliosidosis
	Hypersegmented	Megaloblastic anemia
